# Exogenous H_2_S restores ischemic post-conditioning-induced cardioprotection through inhibiting endoplasmic reticulum stress in the aged cardiomyocytes

**DOI:** 10.1186/s13578-017-0196-9

**Published:** 2017-12-11

**Authors:** Weiming Sun, Jinxia Yang, Yuanzhou Zhang, Yuxin Xi, Xin Wen, Di Yuan, Yuehong Wang, Can Wei, Rui Wang, Lingyun Wu, Hongzhu Li, Changqing Xu

**Affiliations:** 1 Department of Pathophysiology, Harbin Medical University, Baojian Road, Harbin, 150081 China; 2 Department of Pathology, Daqing Medical College, Daqing, China; 3 The Key Laboratory of Cardiovascular Medicine Research (Harbin Medical University), Ministry of Education, Harbin, China; 4 The Cardiovascular and Metabolic Research Unit, Laurentian University, Sudbury, Canada

**Keywords:** Hydrogen sulfide, Post-conditioning, Endoplasmic reticulum stress, Aged cardiomyocytes

## Abstract

**Background:**

A gasotransmitter hydrogen sulfide (H_2_S) plays an important physiological and pathological role in cardiovascular system. Ischemic post-conditioning (PC) provides cardioprotection in the young hearts but not in the aged hearts. Exogenous H_2_S restores PC-induced cardioprotection by inhibition of mitochondrial permeability transition pore opening and oxidative stress and increase of autophagy in the aged hearts. However, whether H_2_S contributes to the recovery of PC-induced cardioprotection via down-regulation of endoplasmic reticulum stress (ERS) in the aged hearts is unclear.

**Methods:**

The aged H9C2 cells (the cardiomyocytes line) were induced using H_2_O_2_ and were exposed to H/R and PC protocols. Cell viability was observed by CCK-8 kit. Apoptosis was detected by Hoechst 33342 staining and flow cytometry. Related protein expressions were detected through Western blot.

**Results:**

In the present study, we found that 30 μM H_2_O_2_ induced H9C2 cells senescence but not apoptosis. Supplementation of NaHS protected against H/R-induced apoptosis, the expression of cleaved caspase-3 and cleaved caspase-9 and the release of cytochrome *c*. The addition of NaHS also counteracted the reduction of cell viability caused by H/R and decreased the expression of GRP 78, CHOP, cleaved caspase-12, ATF 4, ATF 6 and XBP-1 and the phosphorylation of PERK, eIF 2α and IRE 1α. Additionally, NaHS increased Bcl-2 expression. PC alone did not provide cardioprotection in H/R-treated aged cardiomyocytes, which was significantly restored by the supplementation of NaHS. The beneficial role of NaHS was similar to the supply of 4-PBA (an inhibitor of ERS), GSK2656157 (an inhibitor of PERK), STF083010 (an inhibitor of IRE 1α), respectively, during PC.

**Conclusion:**

Our results suggest that the recovery of myocardial protection from PC by exogenous H_2_S is associated with the inhibition of ERS via down-regulating PERK-eIF 2α-ATF 4, IRE 1α-XBP-1 and ATF 6 pathways in the aged cardiomyocytes.

## Background

Hydrogen sulfide (H_2_S) has been recognized as a gasotransmitter with important functions in tissues and organs [1]. Endogenous H_2_S production is mainly catalysed by cystathionine β-synthase (CBS), cystathionine-γ-lyase (CSE) and 3-mercaptosulphurtransferase (3-MPST) [1]. Among them, CSE is a key enzyme in the production of endogenous H_2_S in the cardiovascular system [2–4]. The abnormal metabolism and functions of the CSE/H_2_S pathway have been linked to various cardiovascular diseases, including ischemia/reperfusion (I/R) injury, atherosclerosis, hypertension, heart failure, myocardial infarction etc. [2–5].

I/R (or hypoxia/reoxygenation, H/R) injury increases endoplasmic reticulum stress (ERS) and then induces apoptosis [6]. ERS related proteins, glucose regulated protein 78 (GRP78), C/EBP homologous protein (CHOP) and cleaved caspase-12, and related signaling pathways, pancreatic endoplasmic reticulum kinase (PERK)-eukaryotic translation initiation factor 2α (eIF 2α)-activating transcription factor 4 (ATF 4), inositol-requiring enzyme1α (IRE 1α)- X-box-binding protein-1 (XBP-1) and ATF 6 are increased during I/R (or H/R) [7–10]. As well known that PC inhibits myocardial I/R (or H/R) injury and apoptosis [3, 4]. However, it was recently reported that PC loses its cardioprotection in aged hearts [3, 4, 10–14].

It was reported that H_2_S played an important role by down-regulation of ERS in various systems, especially the cardiovascular system [15–17]. For example, H_2_S suppressed I/R induced-myocardial injury and apoptosis via decrease of ERS [15]. H_2_S had a regulatory role in aortic ERS and reduced atherosclerotic lesions in apoE (−/−) mice fed with a Western type diet [15]. H_2_S attenuated high fat diet-induced cardiac dysfunction via the inhibition of ERS [16]. H_2_S improved vascular calcification in rats by reducing ERS [17].

Our previous studies demonstrated that PC lost cardioprotection, which was related to down-regulation of the CSE/H_2_S pathway in the aged hearts [3, 4, 11–14]. Exogenous H_2_S contributes to recovery of PC-induced myocardial protective effect by decrease of oxidative stress via down-regulation of NF-κB and JAK2-STAT3 pathways, inhibition of mPTP opening via the activation of the ERK1/2-GSK-3β, PI3 K-Akt-GSK-3β and PKC-ε-mitoK_ATP_ pathways and increase of autophagy via up-regulation of AMPK-mTOR pathway [3, 4, 12–14]. However, the involvement in recovery of cardioprotection from PC by exogenous H_2_S via inhibiting ERS is unclear in the aged cardiomyocytes. In order to indicate this question, we used hydrogen peroxide (H_2_O_2_) induced-aged H9C2 cells (the cardiomyocytes line) exposed to PC as an experimental model to demonstrate the effects of exogenous H_2_S on the ERS and relative mechanisms during PC.

## Methods

### Materials

Sodium hydrogen sulfide (NaHS), 4-PBA (an inhibitor of ERS), GSK2656157 (an inhibitor of PERK), STF083010 (an inhibitor of IRE 1α) were purchased from Sigma Chemical Co. (St. Louis, MO, USA). The primary antibodies for anti-Cyclin D1, p21^Cip/WAF−1^, cleaved caspase-3 and -9, Bcl-2, cytochrome *c* (Cyt *c*), GRP 78, CHOP, cleaved caspase-12, ATF 4, ATF 6, XBP-1 and GAPDH were from Proteintech (Wuhan, China). Hoechst 33342 was from Beyotime (Wuhan, China). The anti-PERK, eIF 2α and IRE 1α antibodies were obtained from Cell Signaling Technology (Denver, USA). H_2_O_2_ and PBS solution were from Sigma Chemical Co. (St. Louis, MO, USA). Western blotting relative solutions were from Santa Cruz Biotechnology (Dallas, USA).

### Culture of cardiomyocytes

Rat cardiomyocytes line H9C2 was purchased from the American Type Culture Collection. H9C2 cells were cultured in growth medium DMEM containing 10% fetal bovine serum (FBS), 100 U/ml penicillin, and 100 mg/ml streptomycin. The experiments were performed when the cells reached 70–80% confluence between passages 6 and 10.

### H_2_O_2_ treatment of the aged H9C2 cells

The treatment for H_2_O_2_ induction was as previously described [18]. Briefly, the DMEM supplemented with 10% FBS was removed, and DMEM supplemented with different concentrations (0, 5, 10, 30, 50, 80, 100 μM) H_2_O_2_ was added to the H9C2 cells in the culture cluster for 2 h and subsequently cultured for 3 days. The degree of the aged cells was observed through SA β*-*Gal Staining, AGEs ELISA Assay and the expression of Cyclin D1 and p21^Cip/WAF−1^. In the present study, we selected 30 μM H_2_O_2_ concentrations for 2 h and subsequently cultured for 3 days.

### Hypoxia/reoxygenation (H/R) of the aged H9C2 cells

H/R model of the aged H9C2 cells was established as described previously [3, 4, 12]. A hypoxic condition was produced by D-Hank solution (in mM: 5.37 KCl, 0.44 KH_2_PO_4_, 136.89 NaCl, 4.166 NaHCO_3_, 0.338 Na_2_HPO_4_, 5 d-glucose, pH 7.3–7.4 at 37 °C) saturated with 95% N_2_ and 5% CO_2_. The pH was regulated to 6.8 with lactate to mimic ischemic solution. The aged cardiomyocytes were put into a hypoxic incubator that was equilibrated with 1% O_2_/5% CO_2_/94% N_2_. After hypoxia, the culture medium was rapidly replaced with fresh DMEM with 10% fetal bovine serum (normoxic culture solution) for initiating reoxygenation.

### Experimental protocols of the aged H9C2 cells

**Fig. 1:**
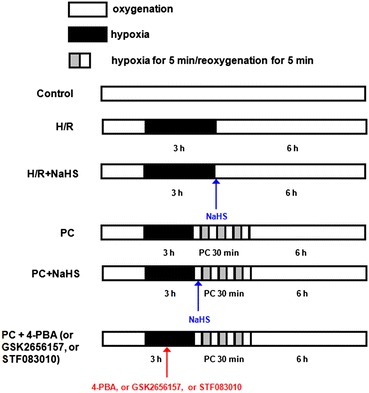
Summary of experimental treatments protocol. The aged H9C2 cells were exposed to hypoxic culture medium for 3 h and reoxygenated for 6 h by replacing the hypoxic culture medium with fresh DMEM with 10% fetal bovine serum. For details of ischemic post-conditioning (PC), NaHS, 4-PBA, GSK2656157 and STF083010 treatments see text

The aged H9C2 cells were randomly divided into the following 6 groups. Each group included 8 samples (n = 8) (Fig. 1): (1) Control group (Control); The aged H9C2 cells were cultured for 9 h with 10% FBS-DMEM; (2) Hypoxia/reoxygenation group (H/R): The aged H9C2 cells were exposed to hypoxic culture medium for 3 h and reoxygenated for 6 h by replacing the hypoxic culture medium with fresh DMEM with 10% FBS; (3) H/R + NaHS group: The procedure was similar to that for group 2, except that 100 μM NaHS were added in 6 h reoxygenation; (4) PC group: At the end of 3 h of hypoxia, the aged H9C2 cells were exposed to normoxic culture solution for 5 min, after which cells were placed in hypoxic solution for 5 min. The PC cycle was repeated three times and followed by 6 h of reoxygenation; (5) PC + NaHS group: At the end of 3 h of hypoxia, initiated immediately at the onset of reoxygenation, 100 μM NaHS were given at the onset of reoxygenation for 5 min following with 5 min hypoxia. This protocol was repeated for another two times. The cells were then treated as those of group 3; (6) PC + 4-PBA (or GSK2656157, or STF083010) group: 0.5 mM 4-PBA (or 10 µM GSK2656157 or 50 µM STF083010) were added to the medium 40 min before the end of hypoxia. The cells were then treated as those of group 4.

### AGEs ELISA assay

The rat advanced glycation end products (AGEs) assay was performed with AGEs ELISA kit according to the instructions from the manufacturer and was as previously described [4, 19, 20]. The reagents of the test kit were placed at room temperature for 30 min and diluted 1:20 with distilled water. Aliquots of 100 µl of the standards and samples were added to blank micropores and 50 µl enzyme marker solution was added. Microtiter plates were incubated at 37 °C for 60 min and then washed five times and put aside for 10–20 s each time. The A and B substrate solutions (50 µl) were added into the microtiter plates for 15 min dark reactions at 37 °C. The reaction was terminated by the addition of 50 µl stop solution, and the optical density (OD) at 450 nm was determined by an ultra microplate reader (Bio-Rad Laboratories, Hercules, CA, USA). An AGEs standard curve was generated and the AGEs values of the samples were calculated from the standard curve.

### SA β-gal staining

Senescence-associated β-gal (SA β-gal) activity was measured with the β-gal staining kit at pH 6.0 according to the instructions from the manufacturer [4, 19, 20]. Briefly, the cells were washed in phosphate buffered saline (PBS), fixed for 10–15 min at room temperature with 1 ml of fixative solution and incubated overnight at 37 °C with the staining solution mix. Cells were observed for development of the blue coloration with a microscope at a magnification of ×400. Aging cardiomyocytes were assessed by counting the number of cells that displayed blue coloration.

### Caspase-3 activity assay

Caspase-3 activity was determined using the ApoAlert Caspase Colorimetric Assay kit in accordance with the manufacturer’s protocol [18]. In brief, at least 1 × 10^6^ cells per sample were lysed in 50 µl of lysis buffer, and the protein concentrations in the samples were estimated using the Bio-Rad protein assay. After incubation on ice for 10 min, the samples were centrifuged at 16,000×*g* for 3 min at 4 °C. Each supernatant was mixed with 50µl 2 × Reaction Buffer/DTT mix and 5 µl of 1 mM Caspase-3 Substrate (DEVD-pNA, 50 µM final concentration), and the samples were then incubated for 1 h at 37 °C in the dark. Developed colour was measured at 405 nm, and caspase-3 activity was calculated in terms of absorbance units per µg protein.

### Cell viability assay

Cell viability was measured by Cell Counting Kit-8 (CCK-8). Cells were seeded in 96-well plates at a concentration of 3 × 10^3^ cells/well. After 24 h of each treatment, 10 μl was added to each well of CCK-8 immediately. Subsequently, they were incubated for 2 h at 37 °C. Using a microplate spectrophotometer, the plates were read at 570 nm (A 570) to determine their optical density.

### Apoptotic rate of cells by Hoechst 33342 staining

Cells were analyzed for apoptosis after visualization of nuclei morphology with fluorescent DNA-binding dye Hoechst 33342, as described previously [4, 12]. After treatment, cells were rinsed with PBS and incubated with 5 µg/ml Hoechst 33342 for 10 min. Nuclei were visualized at 400× magnification using fluorescent microscopy at an excitation wavelength of 330–380 nm. Apoptotic nuclei of cells were assessed by counting the number of cells that displayed nuclear morphology changes, such as chromatin condensation and fragmentation.

### Apoptotic rate of cells by flow cytometry assay

The apoptotic rate was measured by flow cytometry as described previously [4]. Cells were washed three times with ice-cold PBS, and then stained with annexin V-fluorescein isothiocyanate for 15 min at room temperature in 200 μl binding buffer. Next, 300 μl binding buffer was added, and the cells were stained with propidium iodide for 30 min at 4 °C. The fluorescence of the cells was analyzed by flow cytometry. The percentage of apoptotic cells was determined using Mod Fit LT software (Verity Software House Inc., Topsham, ME, USA).

### Western blotting analysis

The related protein expressions were measured by Western blot as described previously [2–6]. Brifely, equal amounts of proteins were subjected to sodium dodecyl sulfate polyacrylamide gel electrophoresis and blotted on polyvinylidene fluoride membranes. The membranes were incubated with primary antibodies. The secondary antibody was goat anti-rat immunoglobulin G. The intensities of the protein bands were quantified by a Bio-Rad ChemiDoc™ EQ densitometer and Bio-Rad Quantity One software (Bio-Rad Laboratories). The protein concentration was quantified using the BCA Protein Assay kit (Beyotime, Nantong, China).

### Detection of Cyt c release from mitochondrial

Western blot analysis of Cyt c in the cytosolic fraction was performed as described previously [3, 4, 12]. Briefly, cells were harvested, washed twice with ice-cold PBS, and incubated in ice-cold Tris-sucrose buffer (0.35 mM sucrose, 10 mM Tris–HCl at pH 7.5, 1 mM EDTA, 0.5 mM dithiothreitol, 0.1 mM phenylmethylsulphonyl fluoride). After a 40 min incubation, cells were centrifuged at 1000×*g* for 5 min at 4 °C and the supernatant was further centrifuged at 40,000×*g* for 30 min at 4 °C. The supernatant was retained as the cytosolic fraction and analyzed by Western blot with a primary rat anti-Cyt c monoclonal antibody and a secondary goat anti-rat immunoglobulin G (Promage). GAPDH expression was used as the control.

### Statistical analysis

All data were expressed as the mean ± SE and represented at least three independent experiments. Statistical comparisons were made using student’s t test or one-way ANOVA followed by a post hoc analysis (Tukey test) where applicable. Significance level was set at p < 0.05.

## Results

### H_2_O_2_ induced senescence in the H9C2 cells

**Fig. 2:**
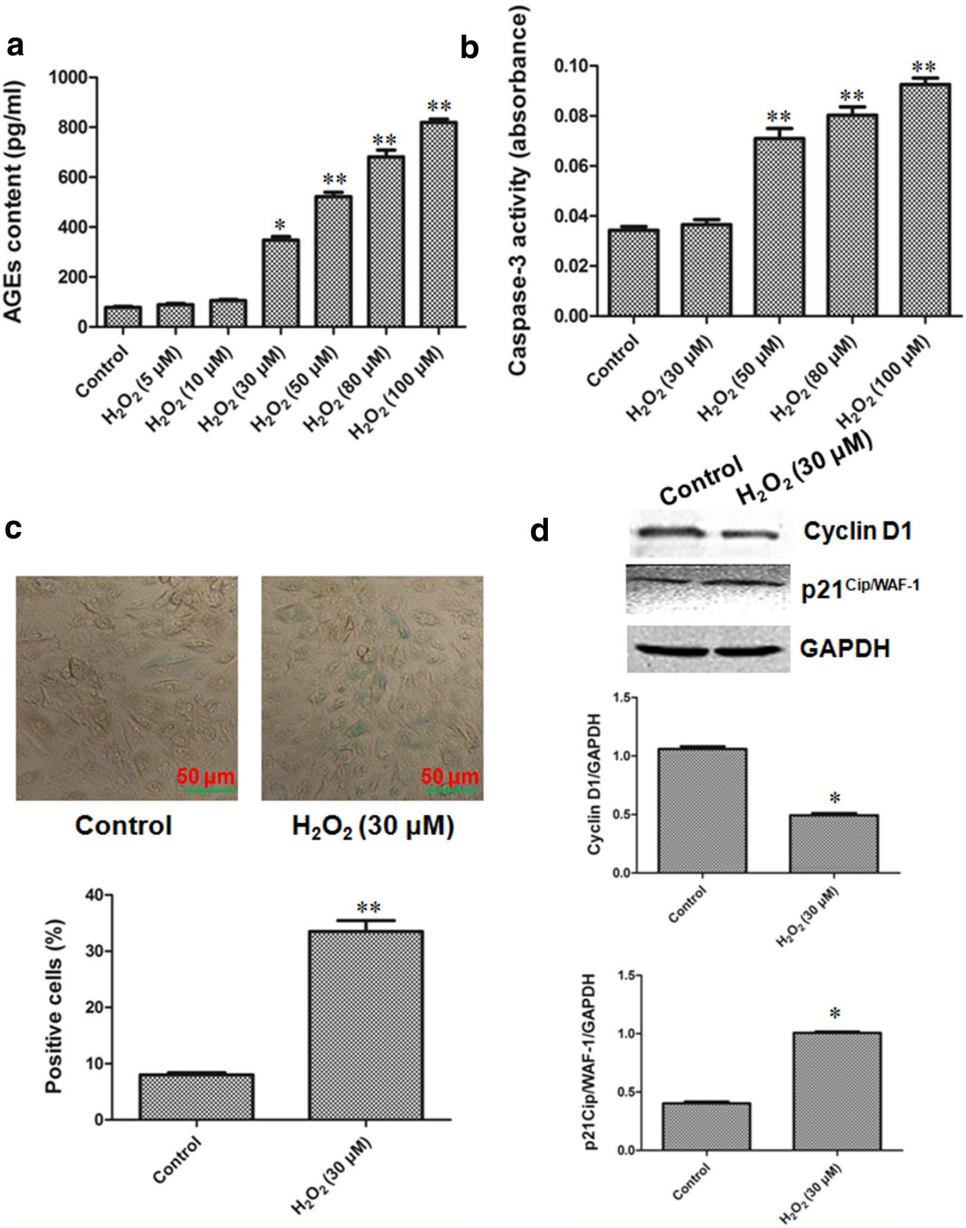
Low dose of H_2_O_2_ induced senescence in the H9C2 cells without causing apoptosis. **a** The AGEs content. The different concentrations (0, 5, 10, 30, 50, 80, 100 μM) H_2_O_2_ was added to the H9C2 cells in the culture cluster for 2 h and subsequently cultured for 3 days. All data were from eight independent experiments. *p < 0.05, **p < 0.01 *vs*. control group. **b** The caspase-3 activity. Caspase-3 activity, measured as luminescence, in the H9C2 cells 3 days after treatment with H_2_O_2_ (30, 50, 80, 100 μM) for 2 h. All data were from four independent experiments. **p < 0.01 *vs*. control group. **c** The number of SA β-gal-positive H9C2 cells. Phase-contrast images showing morphologic changes and stained cells. (SA-β-gal positive cells; Blue, magnification ×400). Scale bar = 50 μm. The aged H9C2 cells in at least five random fields were counted. **p < 0.01 *vs*. control group. **d** The expression of cyclin D1 and p21^Cip/WAF−1^. The intensity of each band was quantified by densitometry, and data were normalized to the GAPDH signal. All data were from four independent experiments. *p < 0.05 *vs*. control group

H_2_O_2_ can induce the senescence of cells by increasing oxidative stress. The increase of AGEs content is an important marker of the senescence. The up-regulation of caspase-3 activity is key marker of the apoptosis. In the present study, the H9C2 cells were exposed to various concentrations of H_2_O_2_ ranging from 0 to 100 μM for 2 h and subsequently cultured for 3 days, after which senescence and apoptosis were determined. The data showed that we successfully induced the senescence of the H9C2 cells with 30 μM H_2_O_2_ and the number of senescent cells was increased in a dose-dependent manner (Fig. 2a), However, it led to apoptosis when exposed to the concentrations of H_2_O_2_ more than 30 μM and 30 μM H_2_O_2_ had no apparent effect on caspase-3 activity (Fig. 2b). To further confirm this phenomenon, we performed SA -Gal staining and the expression of cyclin D1 and p21^Cip/WAF−1^. We found that the number of SA -gal-positive cardiomyocytes and the expression of p21^Cip/WAF−1^ were significantly increased and cyclin D1 expression were markedly decreased after incubation with 30 μM H_2_O_2_ for 2 h and subsequently cultured for 3 days (Fig. 2c, d). Taken together, the concentration of 30 μM H_2_O_2_ induces the senescence but not the apoptosis. Therefore, additional experiments were performed with 30 μM H_2_O_2_.

### H_2_S increased the cell viability

**Fig. 3:**
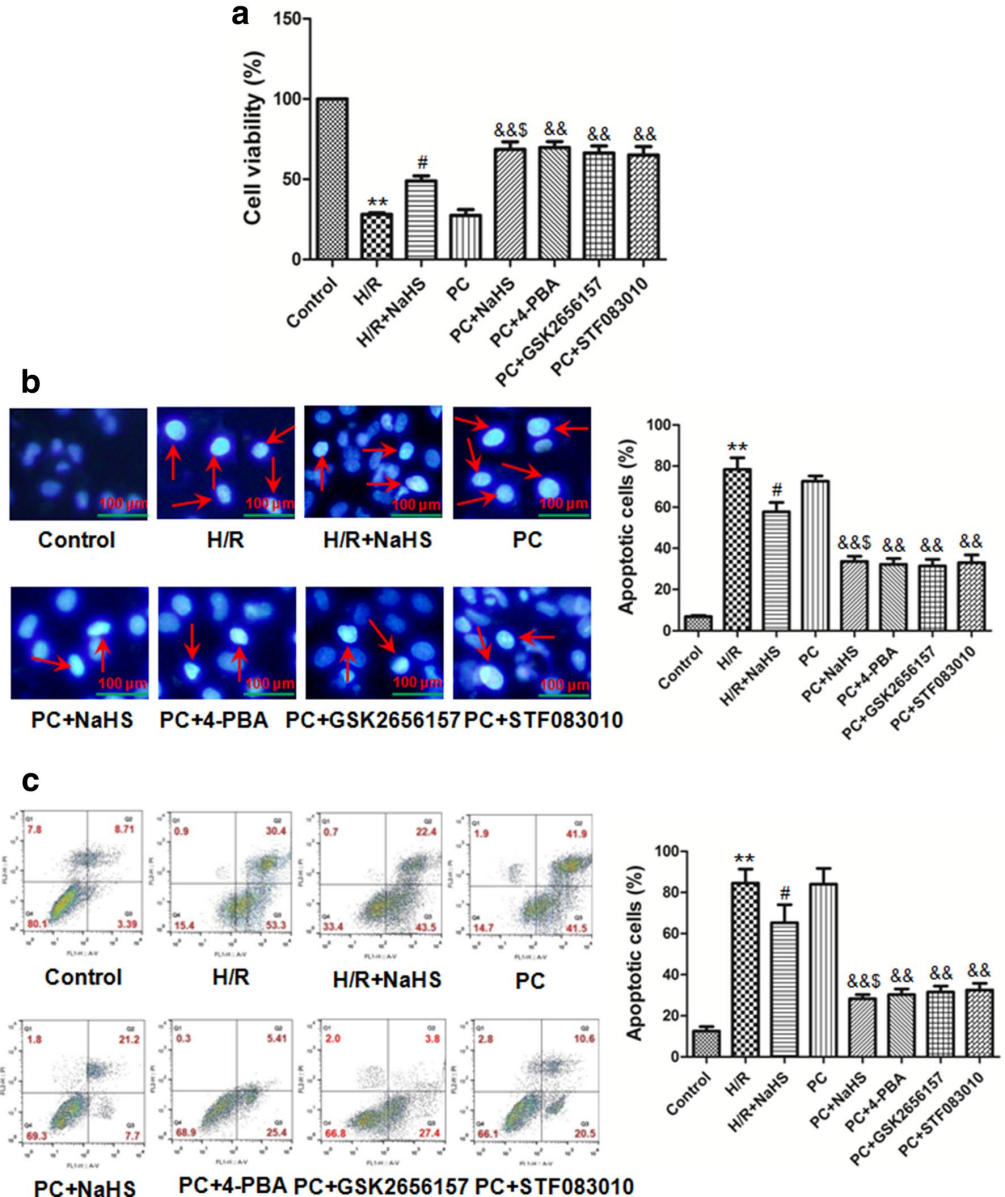
Exogenous H_2_S increased cell viability and decreased apoptotic rate in the aged H9C2 cells. **a** Cell viability was measured by CCK-8 kit. All data are mean ± S.E.M. of 8 determinations. **b** Detection of nuclear morphology in apoptotic cells by Hoechst 33342 staining. Apoptotic cells were identified as cells with condensed, disrupted nuclei. The cells of arrow indicate the apoptotic cells (400× magnification). Scale bar = 100 μm. Apoptotic cells in at least five random fields were counted. **c** Apoptosis analyzed by flow cytometry. The apoptotic rate = early apoptotic rate + late apoptotic rate. All Data were from four independent experiments. **p < 0.01 *vs*. control group; ^#^p < 0.05 *vs*. H/R group; ^&&^p < 0.01 *vs*. PC group; ^$^p < 0.05 *vs*. H/R + NaHS group

Cell viability reflects the condition of cell growth. To assay whether exogenous H_2_S restores PC-induced cardioprotection, we observed the cell viability through CCK8 kit in the H_2_O_2_ induced-aged H9C2 cells. Our results showed that the cell viability was significantly reduced in the H/R group (p < 0.01 versus control group). Compared with the H/R group, the cell viability was increased in the H/R + NaHS group (p < 0.05) and it change was not significant in the PC group (p > 0.05). Compared with the PC group, the cell viability was obviously increased in the PC + NaHS (or 4-PBA, or GSK2656157, or STF083010) (p < 0.01); and the beneficial role of PC + NaHS on the cell viability was similar to PC + 4-PBA (or GSK2656157, or STF083010), respectively. Compared with the H/R + NaHS group, the cell viability was further increased in the PC + NaHS group (p < 0.05) (Fig. 3a). These indicate that exogenous H_2_S restores the PC-induced increase of the cell viability.

### H_2_S inhibited apoptosis

To investigate whether exogenous H_2_S protects against PC-induced cardiac apoptosis, we examined cardiomyocyte apoptosis by Hoechst 33342 staining and flow cytometry. The data showed that H/R significantly increased the apoptotic rate than that in control group (p < 0.01). H/R + NaHS decreased the apoptotic rate (p < 0.05 versus the H/R group). The apoptotic rate of the PC group was similar to the H/R group. Compared with the PC, the apoptotic rate of the PC + NaHS (or 4-PBA, or GSK2656157, or STF083010) was markedly decreased (p < 0.01), and the effect of PC + NaHS on the apoptotic rate was similar to PC + 4-PBA (or GSK2656157, or STF083010), respectively. Compared with the H/R + NaHS group, the apoptotic rate was further decreased in the PC + NaHS group (p < 0.05) (Fig. 3b, c). Our results suggest that exogenous H_2_S restores PC-inhibited apoptosis.

### H_2_S decreased pro-apoptotic factors and increased anti-apoptotic factors

H/R damages mitochondria, and then pro-apoptotic factors (cleaved caspase-3, cleaved caspase-9 and Cyt *c*) and anti-apoptotic factors (Bcl-2) are release from injured mitochondria, which finally lead to apoptosis. The mitochondrial pathway is the most important apoptotic pathway. To further demonstrate exogenous H_2_S restores PC-induced cardioprotection via inhibiting apoptosis, we detected the expression of cleaved caspase-3, cleaved caspase-9 and Bcl-2 in the whole cell lysates as well as Cyt *c* in the cytosolic fraction by western blotting.

**Fig. 4:**
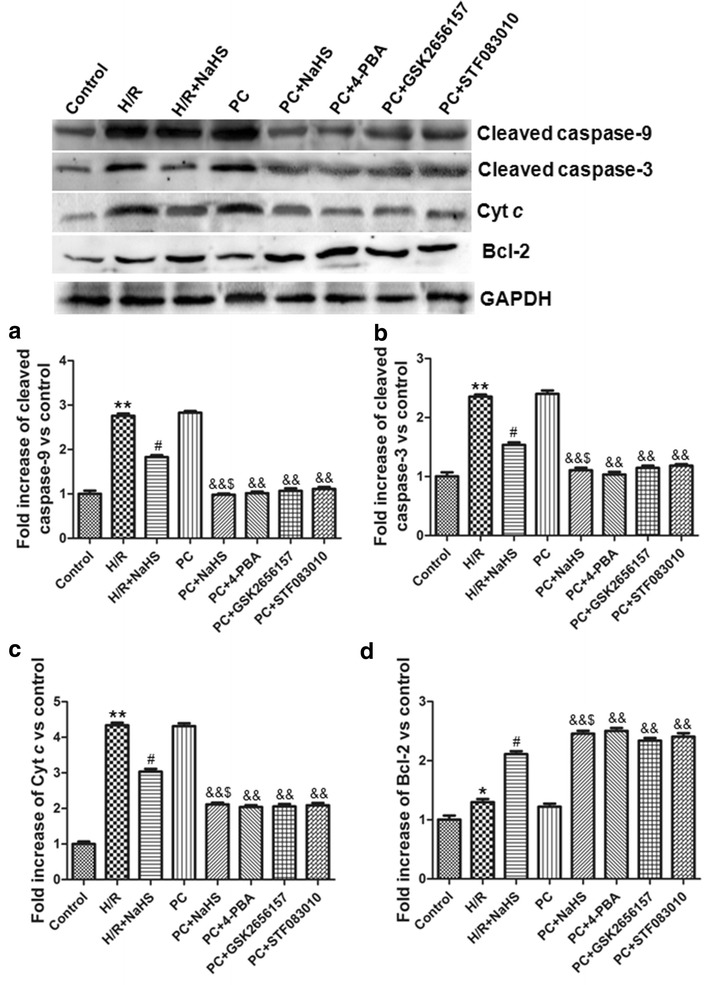
The effect of exogenous H_2_S on the expression of apoptotic relative factors. **a** Cleaved caspase-9; **b** Cleaved caspase-3; **c** Cyt *c*; **d** Bcl-2. The intensity of each band was quantified by densitometry, and data were normalized to the GAPDH signal. The expression levels in the control group were considered the basal levels, and the others are expressed as fold change from the control group. The fold change values represent the mean ± S.E.M. of four determinations. *p < 0.05, **p < 0.01 *vs*. control group; ^#^p < 0.05 *vs*. H/R group; ^&&^p < 0.01 *vs*. PC group; ^$^p < 0.05 *vs*. H/R + NaHS group

Figure 4 showed that the expression of pro-apoptotic factors and anti-apoptotic factors was significantly increased in the H/R group compared with the control group (p < 0.05 or p < 0.01). Compared with H/R, H/R + NaHS decreased expression of pro-apoptotic factors but increased expression of anti-apoptotic factors (p < 0.05). The results of the PC group were similar to those of the H/R group. Compared with the PC, the PC + NaHS (or 4-PBA, or GSK2656157, or STF083010) obviously down-regulated pro-apoptotic factors but up-regulated expression of anti-apoptotic factors (p < 0.01), and the effect of PC + NaHS on these indexes was similar to PC + 4-PBA (or GSK2656157, or STF083010), respectively. PC + NaHS treatment decreased the expression of pro-apoptotic factors and increased the expression of anti-apoptotic factors in comparison with the H/R + NaHS (p < 0.05). These results suggest that exogenous H_2_S restores the anti-apoptotic effect of PC against H/R by preventing the mitochondrial apoptotic pathway (Cyt *c*–caspase-9–caspase-3).

### H_2_S inhibited ERS

**Fig. 5:**
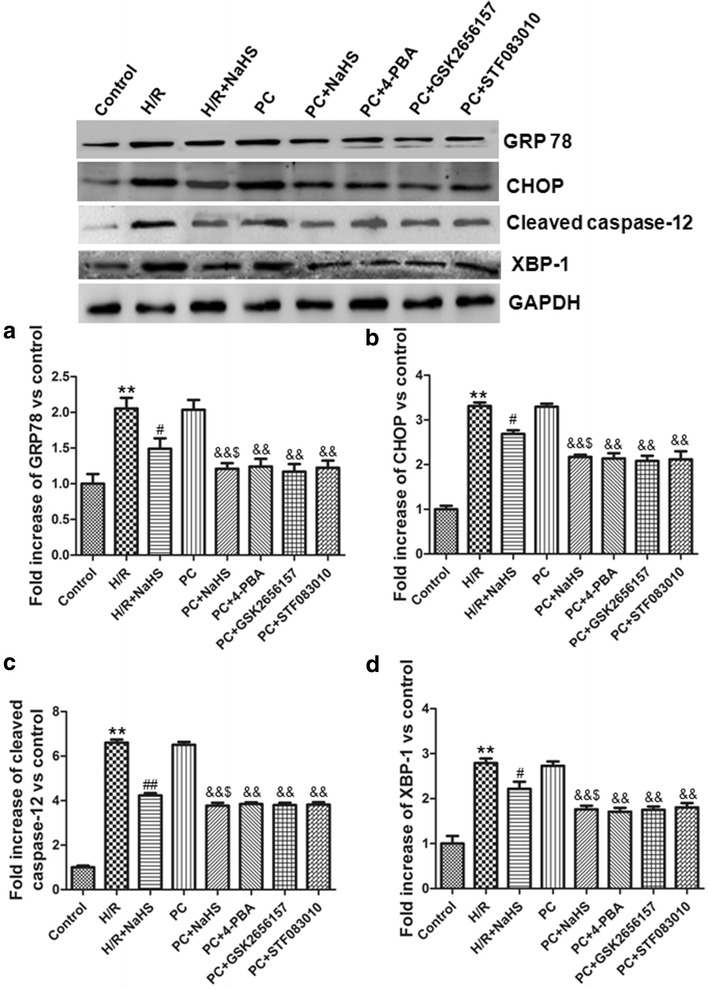
Exogenous H_2_S inhibited the expression of the relative proteins of ERS. **a** GRP 78; **b** CHOP; **c** Cleaved caspase-12; **d** XBP-1. The intensity of each band was quantified by densitometry, and data were normalized to the GAPDH signal. The expression levels in the control group were considered the basal levels, and the others are expressed as fold change from the control group. The fold change values represent the mean ± S.E.M. of four determinations. **p < 0.01 *vs*. control group; ^#^p < 0.05, ^##^p < 0.01 *vs*. H/R group; ^&&^p < 0.01 *vs*. PC group; ^$^p < 0.05 *vs*. H/R + NaHS group

H/R can increase ERS and then induce apoptosis. The relative proteins of ERS mainly include GRP 78, CHOP, cleaved caspase-12 and XBP-1. In this study, we examined the expression of GRP 78, CHOP, cleaved caspase-12 and XBP-1. Our results showed that the expression of GRP 78, CHOP, cleaved caspase-12 and XBP-1 was significantly increased in the H/R group (p < 0.01 versus the control group). The expression of these proteins was decreased in the H/R + NaHS group (p < 0.05 or p < 0.01 versus the H/R group). The change of these indexes in the PC group was similar to that in the H/R group. Compared with the PC, the PC + NaHS (or 4-PBA, or GSK2656157, or STF083010) obviously down-regulated the expression of these proteins (p < 0.01), and the effect of PC + NaHS on these proteins was similar to PC + 4-PBA (or GSK2656157, or STF083010), respectively. Compared with the H/R + NaHS, PC + NaHS further decreased the expression of these proteins (p < 0.05) (Fig. 5), indicating that exogenous H_2_S restores PC-induced myocardial protective effect by inhibiting ERS.

### H_2_S down-regulated PERK-eIF 2α-ATF 4, IRE 1α-XBP-1 and ATF 6 pathways

**Fig. 6:**
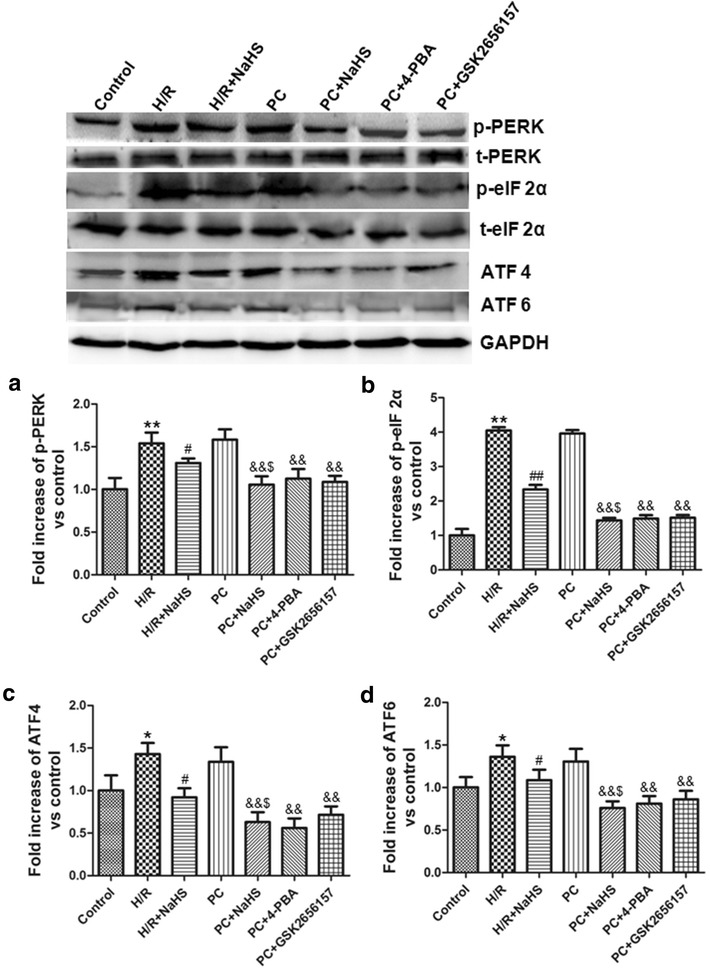
Exogenous H_2_S inhibited PERK-eIF 2α-ATF 4 pathway. **a** PERK; **b** eIF 2α; **c** ATF 4; **d** ATF 6. The graphs represent the optical density of the bands of phosphorylated PERK and eIF 2α normalized with the expression of total PERK and eIF 2α, respectively. The graphs represent the optical density of the bands of ATF 4 and ATF 6 normalized with the expression of GAPDH signal. The expression levels in the control group were considered the basal levels, and the others are expressed as fold change from the control group. The fold change values represent the mean ± S.E.M. of three determinations. *p < 0.05, **p < 0.01 *vs*. control group; ^#^p < 0.05, ^##^p < 0.01 *vs*. H/R group; ^&&^p < 0.01 *vs*. PC group; ^$^p < 0.05 *vs*. H/R + NaHS group

**Fig. 7:**
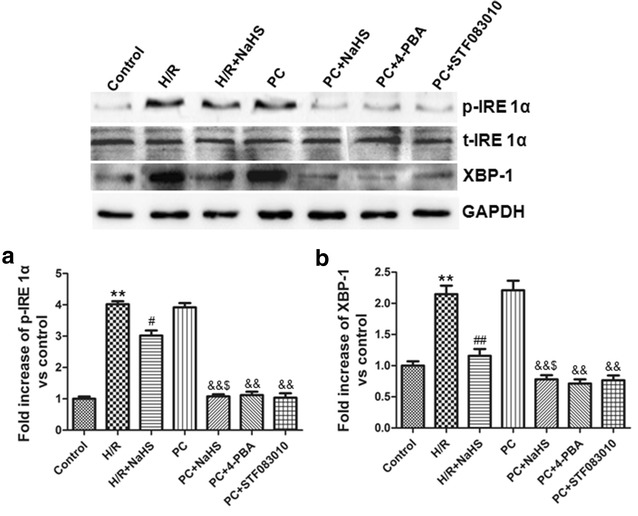
Exogenous H_2_S down-regulated IRE 1α-XBP-1 pathway. **a** IRE 1α; **b** XBP-1. The graphs represent the optical density of the bands of phosphorylated IRE 1α normalized with the expression of total IRE 1α. The graphs represent the optical density of the bands of XBP-1 normalized with the expression of GAPDH signal. The expression levels in the control group were considered the basal levels, and the others are expressed as fold change from the control group. The fold change values represent the mean ± S.E.M. of three determinations. **p < 0.01 *vs*. control group; ^#^p < 0.05, ^##^p < 0.01 *vs*. H/R group; ^&&^p < 0.01 *vs*. PC group; ^$^p < 0.05 *vs*. H/R + NaHS group

PERK-eIF 2α-ATF 4, IRE 1α-XBP-1 and ATF 6 pathways involve in ERS. To detect whether exogenous H_2_S restores PC protective role by inhibiting PERK-eIF 2α-ATF 4, IRE 1α-XBP-1 and ATF 6 pathways, we observed the change of these pathways. The activity of phosphorylated PERK, eIF 2α and IRE 1α and the expression of ATF 4, ATF 6 and XBP-1 in the H/R group were significantly higher than that in the control group (p < 0.05 or p < 0.01). The level of these indexes was lower in the H/R + NaHS group than in the H/R group (p < 0.05 or p < 0.01). The change of these indexes in the PC group was similar to those in the H/R group. Compared with the PC group, the level of these indexes was markedly decreased in the PC + NaHS (or 4-PBA, or GSK2656157, or STF083010) (p < 0.01). The effect of PC + NaHS on PERK-eIF 2α-ATF 4, IRE 1α-XBP-1 and ATF 6 pathways was similar to inhibitor of these pathways, respectively. In the PC + NaHS group, the change of these indexes was decreased to a larger extent than that in the H/R + NaHS (p < 0.05) (Figs. 6, 7). These results demonstrate that exogenous H_2_S restores PC-induced cardioprotection by inhibiting ERS via down-regulating PERK-eIF 2α-ATF 4, IRE 1α-XBP-1 and ATF 6 pathways.

## Discussion

H_2_O_2_ induces senescence by increase of oxidative stress [18]. The increase of AGEs content is one of the most important markers in senescence [21–23]. SA β-gal activity, AGEs content and p21^Cip/WAF−1^ expressions were rised and cyclin D1 expressions were reduced in senescence [4, 21–23]. Our data showed that the number of SA β-gal-positive cells, the AGE content and the expression of p21^Cip/WAF−1^ were increased and cyclin D1 expression was decreased in cardiomyocytes, while the change of caspase-3 activity was not significant, in treated with 30 μM H_2_O_2_ concentrations for 2 h and subsequently cultured for 3 days (Fig. 2). This indicates the successful establishment of H_2_O_2_-induced aged cardiomyocytes model used in the present study.

It is well known that PC inhibits I/R (or H/R) injury and apoptosis [3, 4, 11–14]. However, it was reported that PC loses its myocardial protection in aged hearts [3, 4, 11–14]. The main reason is that aging affects cardiomyocytes at several subcellular and molecular levels, including alterations at the level of the DNA, increased oxidative stress, changes in gene/protein expression and decreased autophagy [3, 4, 11–14]. Our results showed that the difference in cell viability, the apoptotic rate and cleaved caspase-9, cleaved caspase-3, Bcl-2 and Cyt *c* expressions between the H/R and PC groups was not significant in the aged cardiomyocytes (Fig. 3, 4). These results indicate that PC loses protective role against H/R injury in the aged cardiomyocytes. It is consistent with our previous results [3, 4, 12–14].

ERS that disrupts ER function can occur in response to a wide variety of cellular stressors that lead to the accumulation of unfolded and misfolded proteins in the ER [7–10, 24]. Protein markers of ERS mainly include GRP78, CHOP and cleaved caspase-12 [5, 24]. ERS is increased during I/R (or H/R) and promotes apoptosis [6, 18, 24]. In the present study, compared with the H/R group, the cell viability, the apoptotic rate and the expression of cleaved caspase-9, cleaved caspase-3, Cyt *c*, GRP 78, CHOP, cleaved caspase-12 and XBP-1 was significantly decreased and the expression of Bcl-2 was obviously increased in the H/R + NaHS group. Meanwhile, we also found that PC + NaHS further enhanced the cardioprotective roles of H/R + NaHS. The effect of NaHS was similar to 4-PBA (an inhibitor of ERS), GSK2656157 (an inhibitor of PERK), STF083010 (an inhibitor of IRE 1α) during PC, respectively (Figs. 3, 4, 5). These data suggest that exogenous H_2_S restored PC-induced cardioprotection by inhibition of ERS in the aged cardiomyocytes.

ERS includes three signaling pathways: PERK, IRE 1 and ATF 6 [5, 6, 25]. PERK is an ERS sensor and a serine threonine kinase that phosphorylates eukaryotic translation initiation factor 2α (eIF 2α) on ERS [6, 26]. The phosphorylated eIF 2α induces the expression of ATF 4, and then increases CHOP [6]. In mammalian cells, the substrate for the IRE1α endoribonuclease is the XBP-1 mRNA [7, 27]. After activation, the IRE1α endoribonuclease splices the XBP-1 mRNA with an altered reading frame. Activated IRE1α- XBP-1 pathway up-regulates the GRP 78, CHOP and cleaved caspase-12, and then promotes apoptosis [7, 25]. ATF 6 is an ER transmembrane protein [8]. After cleavage, the cytosolic N-domain of ATF 6 translocates into the nucleus where it induces the expression of GRP78, CHOP and XBP-1 [9, 26]. Our data showed that H/R + NaHS decreased PERK-eIF 2α-ATF 4, IRE 1α-XBP-1 and ATF 6 pathways. PC + NaHS further enhanced the effects of H/R +NaHS. And the effect of NaHS on PC was similar to 4-PBA, GSK2656157, STF083010 during PC, respectively (Figs. 6, 7). Taken together, these findings suggest that exogenous H_2_S plays an important role in the recovery of PC-induced cardioprotection by down-regulation of ERS via the inhibiting PERK-eIF 2α-ATF 4, IRE 1α-XBP-1 and ATF 6 pathways in the aged cardiomyocytes.

## Conclusion

**Fig. 8:**
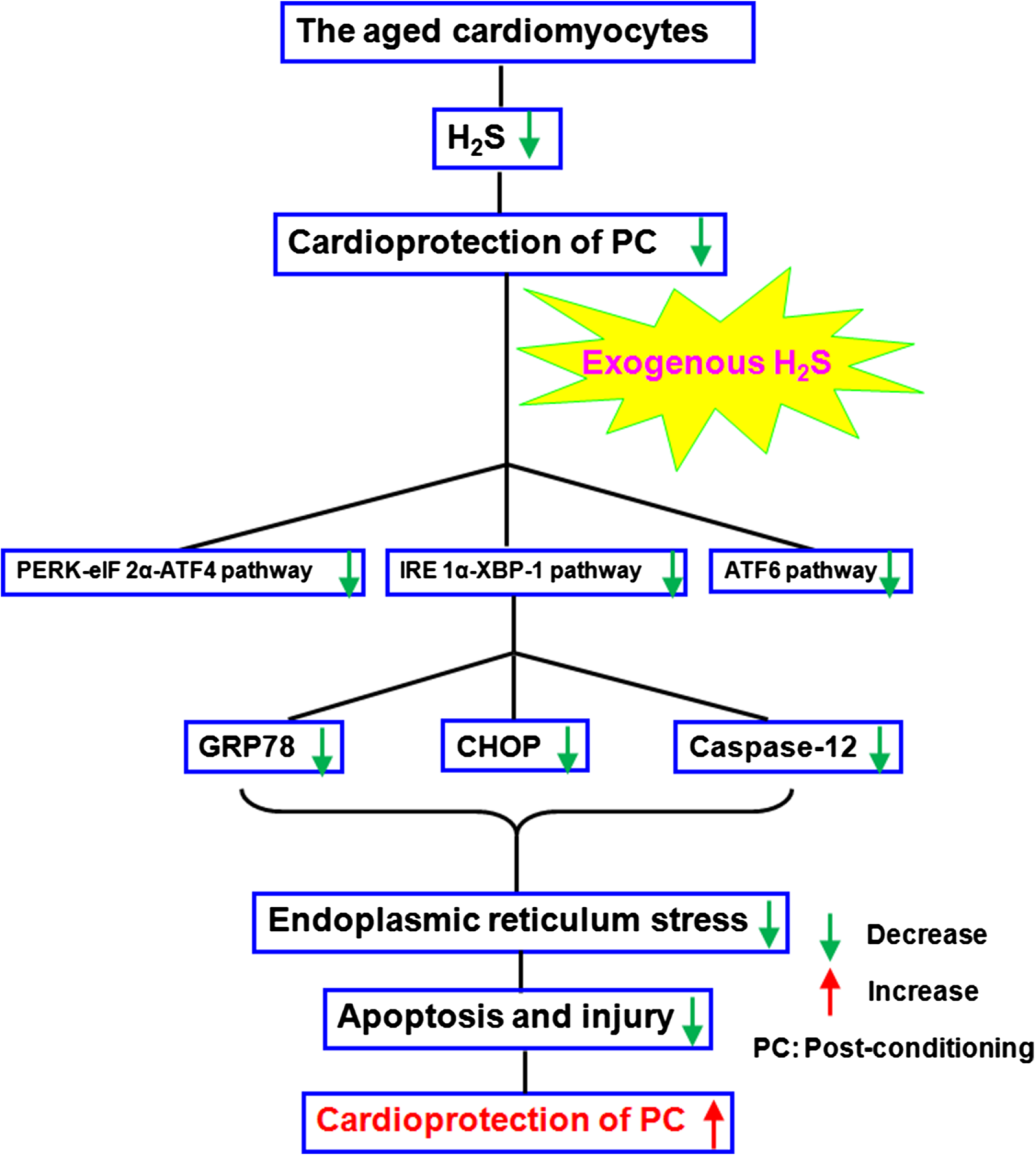
Exogenous H_2_S restores PC-induced cardioprotection in the aged cardiomyocytes. Exogenous H_2_S contributes to recovery of PC-induced cardioprotection by down-regulation of endoplasmic reticulum stress via inhibition of PERK-eIF 2α-ATF 4, IRE 1α-XBP-1 and ATF 6 pathways in the aged cardiomyocytes

In conclusion, our present study demonstrates that the recovery of PC-induced cardioprotection by exogenous H_2_S is associated with the inhibition of ERS through down-regulating PERK-eIF 2α-ATF 4, IRE 1α-XBP-1 and ATF 6 pathways in the aged cardiomyocytes. These findings provide new insight into the prevention and therapy of aged ischemic cardiomyopathy (Fig. 8).
